# Pain in Breast Cancer Treatment: Aggravating Factors and Coping Mechanisms

**DOI:** 10.1155/2014/832164

**Published:** 2014-10-01

**Authors:** Maria de Fatima Guerreiro Godoy, Livia Maria Pereira de Godoy, Stelamarys Barufi, José Maria Pereira de Godoy

**Affiliations:** ^1^Medicine School of São Jose do Rio Preto (FAMERP), Research Group of Godoy Clinic, Avenida Constituição 1306, 15025-120 São Jose do Rio Preto, SP, Brazil; ^2^Medicine School of Santos, UNILUS and Research Group of Godoy Clinic, 15025-120 São Jose do Rio Preto, SP, Brazil; ^3^Lymphovenous Rehabilitation (FAMERP) and Research Group of Godoy Clinic, 15025-120 São Jose do Rio Preto, SP, Brazil; ^4^Cardiovascular Surgery Department, Medicine School in São José do Rio Preto (FAMERP) and National Council for Research and Development (CNPq), 15025-120 São Jose do Rio Preto, SP, Brazil

## Abstract

The objective of this study was to evaluate pain in women with breast cancer-related lymphedema and the characteristics of aggravating factors and coping mechanisms. The study was conducted in the Clinica Godoy, São Jose do Rio Preto, with a group of 46 women who had undergone surgery for the treatment of breast cancer. The following variables were evaluated: type and length of surgery; number of radiotherapy and chemotherapy sessions; continued feeling of the removed breast (phantom limb), infection, intensity of pain, and factors that improve and worsen the pain. The percentage of events was used for statistical analysis. About half the participants (52.1%) performed modified radical surgery, with 91.3% removing only one breast; 82.6% of the participants did not perform breast reconstruction surgery. Insignificant pain was reported by 32.60% of the women and 67.3% said they suffered pain; it was mild in 28.8% of the cases (scale 1–5), moderate in 34.8% (scale 6–9), and severe in 4.3%. The main mechanisms used to cope with pain were painkillers in 41.30% of participants, rest in 21.73%, religious ceremonies in 17.39%, and chatting with friends in 8.69%. In conclusion, many mastectomized patients with lymphedema complain of pain, but pain is often underrecognized and undertreated.

## 1. Introduction

Chronic pain affects from 25% to 60% of patients submitted to breast cancer treatment, and thus this is an important clinical problem which involves intra- and postoperative factors [1]. Chemotherapy is associated with more severe and more persistent physical symptoms and thus patients should receive appropriate care to mitigate the effects [2]. Fatigue is higher in patients with signs and symptoms such as neck pain, shoulder pain, reduced mobility, and bad body image, demonstrating a multidimensional character that affects psychological and physical aspects of the patient's life [3]. Studies have shown that patients submitted to breast cancer surgery may have central hypersensitivity involving painful symptoms of the neck, axilla, and shoulder [4]. The type of surgery, the lack of a spouse, postoperative infection, the number of lymph nodes resected, radiation therapy, high body mass index (BMI), low socioeconomic level, and advanced disease are associated with pain [5]. Nerve injuries due to surgical trauma and the formation of neuromas are associated with postoperative pain after breast cancer [6]. In relation to the forms of treatment, one study reported that 22.3% of 461 patients with shoulder bursitis were successfully treated with local therapy on the medial border of the scapula [7]. However, a meta-analysis was unable to confirm that transcutaneous electrical stimulation of the nerve is an efficacious form of treatment for pain in these patients [8]. Women have reported that yoga can improve several domains of their quality of life during chemotherapy. This mind-body intervention may help, but more randomized controlled trials about the effects of yoga on the cognition of women with breast cancer under adjuvant chemotherapy treatment should be carried out [9].

The objective of this study was to evaluate pain in women with breast cancer treatment-related lymphedema and the characteristics of aggravating factors and coping mechanisms.

## 2. Methods

Descriptive, qualitative study assessing pain reported after treatment of breast cancer surgery in a group of 46 women.

All patients who had been subjected to breast cancer treatment and who were in rehabilitation due to lymphedema in the Clinica Godoy in São Jose do Rio Preto, SP, in 2010 were considered for participation in this study.

The following variables were investigated using a questionnaire: type and length of surgery, number of radiotherapy and chemotherapy sessions, infection, pain (at removed breast or at another site of the body), intensity of pain, and factors that improved and worsened the pain. The intensity of the pain was evaluated using a visual scale of 0 to 10 (No pain: 0; Mild pain: 1 to 5; Moderate pain: 6 to 9; Severe pain 10). The body weight and height were measured to calculate the BMI. The responses to each item were analyzed as a frequency of events (%) and mean or median for age.

All patients who accepted to participate in the study signed informed consent forms and the study was approved by the Research Ethics Committee of FAMERP (Protocol number 85923).

## 3. Results

About half (52.1%) of the participants performed modified radical surgery (91.3% unilateral and 8.6% bilateral) and only 17.37% underwent breast reconstruction surgery; the surgery was performed after more than 19 months in almost 74% of cases. Six to ten sessions of chemotherapy were used in 63.04% of the cases and more than 30 sessions of radiotherapy in 71.3% in a period from 1 to 120 months were used (Table 1).

Insignificant pain was reported by 32.61% of the women and 67.3% said they suffered pain; it was mild in 28.26% of the cases (scale 1–5), moderate in 34.78% (scale 6–9), and severe in 4.35% (Table 2).

The main coping mechanisms were painkillers for 41.30%, rest for 21.74%, participating in religious services for 17.39%, and chatting with friends for 8.70% (Table 2).

Another characteristic was that 39.1% of the women reported that they had pain outside the region of the surgery (such as pain in the body and neck, headaches, etc.) within 6 to 12 months after the surgery which reduced after two years. At the site of the breast, 39.13% reported that the pain persisted for up to one year and 4.34% for up to two years (Table 2). Worsening of the pain with physical effort was reported by 50% of the cases, with stress by 34.8%, heat by 13.0%, and cold by 2.2% (Table 2).

## 4. Discussion

This study shows the prevalence of pain, the coping mechanisms, and the aggravation factors in women treated for breast cancer and subsequently for lymphedema. Pain was reported by 67% of the study sample, with varying intensities of pain and using different mechanisms for its control. This prevalence is similar to that reported in the literature [1].

The use of painkillers was the most cited mechanism, followed by resting. However, unconventional options were also reported as a mechanism of coping with pain such as participating in religious services as described by 17.3% of the patients. Thus, a search for something higher and spiritual constituted a protective mechanism against physical pain. Another aspect that calls the attention is that more than 52% of these patients were college graduates and that the level of education did not interfere in their pursuit for faith as a mechanism of pain control. Several factors are cited that can affect pain such as psychological aspects, infection, immunotherapy, type of surgery, nerve injuries, and bursitis; [1–8] an association of these factors may aggravate the patient's status even further.

Another way of controlling pain was chatting with friends as mentioned by 8.6% of the women; thus, a mechanism for pain control that involved spiritual or psychological acts was used by more than 25% of the cases.

An important differential that caught the attention was in relation to pain and its location either at the site of surgery or in other parts of the body. The reports of pain in other locations were associated with emotional aspects associated with treatment and sometimes with the type of surgery used. However, the reduction of pain over time was important in the evolution of patients.

Worsening of pain on physical exertion was reported by 50% of the patients, with stress being the second most common factor in 34.7% of patients. Environmental factors, such as heat and cold, also influenced the symptoms.

Thus, several factors that interfere in the pain were identified by participants as were the mechanisms (physical, psychological, religious, and environmental) they used to control it. These methods of coping with the pain help patients to alleviate their suffering. In addition to the physical and psychological aspects, a broad approach to these patients involves an investigation of difficulties experienced in their day-to-day lives, coping mechanisms, nutritional aspects, and the adaption to their reality; this approach must be individualized [9–12].

In respect to the occupation of participants in this study, many reported that the surgery took place after they had retired; a second group requested retirement due to illness. The retired individuals had activities in their homes and remained with their families and those who had requested sick leave, even after two years, did not return to their normal activities as they reported feeling a loss of the physical functioning of the arm on the operated side.

The majority of individuals who continued in their jobs were self-employed in informal activities and without the necessary documentation to claim sick allowance and thus could not stop working. The majority of participants had completed college education, but this did not change their psychological, social, and physical difficulties. Most did not receive psychological support or any type of physical therapy after surgery.

These data highlight the multifactorial consequences of breast cancer treatment, which often are associated and are difficult to detect and so it is difficult to give the required support.

In this study, it was found that most of the participants did not receive any kind of psychological and physical support from different health services during treatment. Treatment programs that involve a multidisciplinary team and that more broadly address the problems of these patients are thus recommended [13–15] and pain should always be taken into account during treatment.

The results of this study show the need for rehabilitation programs immediately after breast cancer surgery. It is being recognized by more and more health care professionals that morbidity after breast cancer treatment causes functional sequelae that interfere in the daily life of patients [13, 14].

## 5. Conclusion

In conclusion, many mastectomized patients with lymphedema complain of pain, but pain is often underrecognized and undertreated.

## Figures and Tables

**Table 1:** Time between breast cancer surgery and reconstruction surgery.

Time after surgery	*n*	%
1 to 6 months	5	10.87
7 to 18 months	7	15.22
19 to 24 months	10	21.74
25 to 48 months	14	30.43
60 to 120 months	10	21.74

**Table 2:** Coping mechanisms, aggravating factors, and pain intensity.

	*n*	%
Coping mechanism		
Leisure	5	10.87
Religious service	8	17.39
Chatting with friends	4	8.70
Painkillers	19	41.30
Rest	10	21.74
Aggravating factors		
Physical effort	23	50.00
Stress	16	34.78
Heat	6	13.04
Cold	1	2.17
Pain persisted		
<1 year	18	39.13
>1 year	2	4.34
Pain intensity		
None (0)	15	32.61
Mild (1–5)	13	28.26
Moderate (6–9)	16	34.78
Severe (10)	2	4.35
